# Developmental Switch From Elemental to Configural Processing of Odor Mixtures in Humans

**DOI:** 10.1002/dev.70188

**Published:** 2026-08-05

**Authors:** Sébastien Romagny, Gérard Coureaud, Thierry Thomas‐Danguin

**Affiliations:** ^1^ Université Bourgogne Europe, Institut Agro, CNRS, INRAE, UMR CSGA, 21000 Dijon France; ^2^ Centre de Recherche en Neurosciences de Lyon, Equipe NeuroEthologie Sensorielle, CNRS UMR 5292 Inserm, Université Claude Bernard Lyon 1, Université Jean‐Monnet Saint‐Etienne, 69500 Bron France

**Keywords:** adolescents, adults, children, olfaction, olfactory processing, perception, sensory development

## Abstract

Human perceptual abilities evolve throughout development, particularly with regard to processing complex stimuli. While many studies have investigated age‐related changes in olfactory sensitivity, none have examined how developmental stages influence the perception of odor mixtures at the elemental and configural levels. In this study, we evaluated how children, teenagers, and adults (mean ages: 10, 15, and 39 years) perceive odor mixtures known to trigger either elemental or configural processing in adults. Two binary mixtures (AB and AB′) and two senary mixtures (RC and RC′), known to elicit distinct perceptual strategies in adults, were evaluated using three tasks: typicality rating, target detection, and complexity evaluation. Results showed that perception of the senary mixtures remained consistent across age groups, indicating stable processing of these complex mixtures. In contrast, age‐related differences emerged for the binary mixtures. Children perceived AB as less complex than AB′, but did not rate it as more typical of the configural odor, whereas adults rated AB as more typical of this odor than AB′ and tended to show reduced access to mixture components in binary mixtures, with teenagers displaying an intermediate profile. Overall, these findings suggest developmental changes in odor mixture processing, consistent with a progressive shift from more elemental to more configural olfactory strategies, particularly for mixtures of lower chemical complexity.

## 1 Introduction

As soon as their sensory systems become functional, organisms must be able to extract meaningful information from their environment. Relevant environmental cues can be learned prenatally, stored, and subsequently used to guide appropriate behaviors postnatally. Such processing may further evolve throughout development as a result of learning, changing environmental demands, and the maturation of cognitive and perceptual systems. This is the case with olfaction, for example, as the olfactory system develops and begins functioning before birth in many species, including mammals (e.g., Gottlieb 1971, 1983; Schaal 2000; Coureaud et al. 2002; Romagny et al. 2012).

Olfaction involves organisms processing stimuli in the form of volatile molecules, which usually emanate from sources as mixtures of several odorants rather than as single‐molecule stimuli. Therefore, from early life through to adulthood, organisms primarily process odor mixtures. Understanding how such mixtures are perceptually processed is thus critical for characterizing olfactory function in ecologically valid conditions. Odor mixtures are not perceived as the simple sum of their components; they can be processed elementally, when the mixture is perceived through its individual components, or configurally, when the percept differs from the components, either due to overshadowing among odorants or the emergence of a novel odor quality (Kay et al. 2005; Thomas‐Danguin et al. 2014). In this framework, configural processing refers to the emergence of a unified percept that is not reducible to its individual components, whereas elemental processing refers to access to these components.

Over the past two decades, studies of configural and elemental processing of odor mixtures have been conducted in human adults and newborn rabbits (see also Coureaud et al. [2022], for comparative results in rodents and bees) using two model mixtures of different complexity. In both species, these mixtures, namely a binary pineapple‐odor‐eliciting mixture (AB; Thomas‐Danguin et al. 2007; Le Berre, Béno, et al. 2008; Barkat et al. 2012) and a senary Red Cordial‐odor‐eliciting mixture (RC; Le Berre et al. 2010; Sinding et al. 2013), can be perceived either configurally or elementally (Coureaud et al. 2008, 2009; Coureaud, Thomas‐Danguin, Datiche, et al. 2014; Coureaud, Thomas‐Danguin, Wilson, et al. 2014; Sinding et al. 2013; Romagny et al. 2014), with the mode of perception influenced by prior exposure and learning (Le Berre, Thomas‐Danguin, et al. 2008; Le Berre et al. 2010; Sinding et al. 2011). While human adults tend to perceive a specific configural quality (e.g., pineapple or red cordial) through robust configural processing, newborn rabbits display weak configural processing, detecting both mixture‐specific and component‐related cues. Following previous definitions (Kay et al. 2005; Thomas‐Danguin et al. 2014), robust configural processing refers to the perception of the mixture‐specific (configural) quality with limited access to the qualities of individual components, whereas weak configural processing refers to the perception of both the configural quality and the qualities of the individual components.

The contrast between robust configural perception in human adults and weak configural perception observed for the same mixture in newborn rabbits may reflect species differences or variations in developmental stage. A developmental study in young rabbits showed that the perception of the AB mixture shifts from weak configural to robust configural processing by Day 9, when they no longer detect the components’ odors (Coureaud et al. 2020). This transition occurs during a period of rapid maturation of sensory and perceptual functions, suggesting that developmental changes in these processes may contribute to the emergence of configural processing. Therefore, configural processing may develop progressively from birth to weaning in rabbits, at least for certain mixtures.

This developmental trend is consistent with broader evidence from both human and non‐human species showing that perceptual strategies for processing complex odor stimuli are plastic and may shift from elemental to more configural processing (Derby et al. 1996; Livermore and Laing 1996; Chandra and Smith 1998; Deisig et al. 2001; Wilson and Stevenson 2003; Rokni and Murthy 2014). In humans, however, the time course of such a transition remains unclear. Given that learning and experience can modulate the processing of odor mixtures (Livermore and Laing 1996; Sinding et al. 2015), these perceptual strategies may evolve across development alongside the maturation of cognitive and perceptual processes. Such developmental modulation has been documented in humans for visual stimuli, with studies consistently showing that configural processing develops progressively with age, emerging between infancy and adolescence (Younger and Cohen 1986; Mondloch et al. 2002; Cohen Kadosh et al. 2013). These findings, largely demonstrated in face perception, are consistent with a shift from feature‐based to more integrative processing strategies. Although face perception represents a specialized and socially relevant domain, this shift is generally interpreted as reflecting a broader change in perceptual organization, from the processing of individual features to their integration into a unified percept. Taken together, these findings suggest that perceptual strategies may evolve progressively during development, with childhood and adolescence representing relevant periods to investigate transitions from more elemental toward more configural forms of perception. Nevertheless, such developmental changes have not yet been investigated for odor mixture perception in humans.

To address this gap, the present study investigated how odor mixture processing may change across age in humans. Based on previous developmental findings in rabbits and on evidence from visual perception, we hypothesized that younger participants would show greater access to mixture components and weaker configural perception than older participants. We compared the AB and RC mixtures known to be configurally perceived by adults with two analogous mixtures differing in component proportions (AB′ and RC′), known to be elementally perceived. Children, teenagers, and adults were tested using three tasks divided into two experiments. These tasks were selected because they provide complementary indices of perceptual processing: some tasks are expected to favor access to individual components, whereas others promote the perception of the mixture as a whole. As such, they are expected to provide indirect indices of elemental and configural percepts, rather than direct measures of these processes. In a first experiment, participants rated the perceptual complexity of the mixture; we expected elemental mixtures to be perceived as more complex due to the detection of multiple components. In a second step, a typicality rating task assessed how strongly the mixtures evoked the target configural odors (pineapple or red cordial); higher typicality ratings were expected for configural mixtures, as this task has been shown to favor configural processing (Le Berre, Thomas‐Danguin, et al. 2008). A second experiment involved a detection task designed to promote access to individual odor qualities and, therefore, elemental processing. We hypothesized that participants would show reduced access to individual odor qualities in configurally than in elementally perceived mixtures.

## 2 Material and Methods

### 2.1 Participants

The study was conducted during two sessions of the Experimentarium, a science outreach event organized by the Vulgarization Unit of the University of Burgundy in Dijon, France. The event enables people of all ages to interact with research laboratories and experiments. No a priori power analysis was performed. The sample size was determined by the number of participants that could be recruited during the public event, with the aim of maximizing inclusion across age groups. This resulted in relatively large samples, allowing robust statistical analyses of the main effects and interactions of interest. A total of 376 participants took part: 259 in Experiment 1 and 117 in Experiment 2. In Experiment 1, participants were divided into three age groups: children (*n* = 70, 35 girls, 35 boys), teenagers (*n* = 106, 62 girls, 44 boys), and adults (*n* = 83, 43 women, 40 men). The children were aged between 9 and 11 (10.2 ± 0.4), the teenagers between 14 and 17 (15.7 ± 1.2) and the adults between 18 and 71 (43.6 ± 14.8). Experiment 2 included only children (*n* = 48, 29 girls, 19 boys of 10.0 ± 0.6 years old) and adults (*n* = 69, 42 women, 25 men of 39 ± 11 years old) who had not participated in Experiment 1.

Participants reported no issues related to smell or allergies and were instructed to avoid smoking, drinking, and eating for at least 1 h prior to each session. They were also asked to avoid wearing perfume. As this was an outreach study, participants were not compensated for their participation. All participants signed an informed consent form, although the study's specific aims were not disclosed beforehand. The experiments complied with ethical standards required by French law, and the protocol was reviewed and approved by the Human Research Ethics Committee and the French National Agency for Medicines and Health Products Safety (Dijon, France; # 2012‐A00959‐34).

### 2.2 Stimuli

Four mixtures were used in the study. Two of these are known to be perceived configurally by adults: the binary “AB” mixture (pineapple) and the senary “RC” mixture (red cordial) (Le Berre, Thomas‐Danguin, et al. 2008). These mixtures were formulated by a flavorist to produce specific odor qualities that are distinct from those of their components. The AB mixture contained ethyl isobutyrate (2.37 × 10^−2^ g·mL^−^^1^; CAS # 97‐62‐1) and ethyl maltol (0.55 × 10^−3^ g·mL^−^^1^; CAS # 4940‐11‐8). All the odorants were food grade and were purchased from Sigma‐Aldrich (Saint‐Quentin Falavier, France). The odorant solutions were prepared in ethanol (anhydrous, 99.9% Carlo Erba, France). Using the same two components, another mixture, AB′, was tested. This mixture was known to be elementally perceived when the respective concentrations of A and B are 5.5 × 10^−2^ and 0.237 × 10^−3^ g·mL^−^^1^ (Sinding et al. 2013). When sampled individually, A and B were used at a concentration of 10%. At these concentrations, all stimuli were considered to be iso‐intense (Sinding, 2012).

The RC and RC′ mixtures were the other two odor stimuli and are known to be perceived configurally and elementally, respectively. RC contained six components: vanillin (V, CAS # 8014‐42‐4), frambinone (F, CAS # 5471‐51‐2), isoamyl acetate (IA, CAS # 123‐92‐2), β‐ionone (B, CAS # 79‐77‐6), ethyl acetate (EA, CAS # 141‐78‐6), and damascenone (D, CAS # 23696‐85‐7). Stock solutions of Odorants V, F, IA, D, B, and EA were prepared in ethanol (anhydrous, 99.9%) at 10, 10, 1, 1, 1, and 10% (w/w), respectively. The stock solutions were mixed to obtain final ethanol concentrations of 16.2, 16.2, 1.9, 1.1, 1.6, and 1.6 × 10^−3^ g/ml for V, F, IA, D, B, and EA, respectively. RC′ was an elemental mixture containing the same odorants all at 16.7% ratio.

To deliver odor stimuli to participants, 20 µL of each mixture was applied to four strips of filter paper (40 mm × 5 mm). The ethanol was allowed to evaporate for 2 min under a constant airflow hood, after which the strips were immediately placed in a 60 mL brown glass vial. The vials were then stored at room temperature (21 ± 1°C) for 36 h to allow the gas phase to reach equilibrium before testing. During an experiment, each vial was opened no more than 10 times, with at least a 1‐h interval between participants to allow re‐equilibration of the gas phase. We internally verified that repeated openings did not affect perceived intensity. Each vial was labeled with either a three‐digit random number or a label enabling participants to identify targets (see the procedure below for details).

### 2.3 Experimental Procedure

Tests were conducted in a quiet air‐conditioned meeting room (21 ± 1°C) with groups of eight participants. Each participant sat at a desk facing a white wall to minimize distraction.

In Experiment 1, participants were given a paper questionnaire (see below) and four brown vials containing the four mixtures in a pseudo‐random order. The vials containing the AB or RC mixtures were always presented first, followed by the AB′ and RC′ mixtures, to prevent participants from gaining prior experience of the mixture components that might favor elemental perception (Le Berre et al. 2010). Participants first smelled either the AB or RC mixtures in a random order, then the AB′ or RC′ mixtures. The questionnaire contained 15 pages, with each page corresponding to one question. First, participants evaluated the complexity of the mixture by answering “How many odors did you smell?” with the possible responses being “no odor,” “one odor,” or “more than one odor.” The second question assessed the typicality of the perceived odor compared to a target reference (pineapple or red cordial): “Does this odor remind you of [reference name]?” The possible answers were “not at all,” “a little,” “moderately,” “a lot,” or “tremendously”. As a control, the same question was asked using an unrelated reference label (“mint” for AB and AB′, “lemon” for RC and RC′) to evaluate response reliability. The order in which the target and control questions were presented was randomized across questionnaires. After completing these evaluations, participants also rated their familiarity with pineapple or red cordial using a five‐point scale (“*never eat/drink*,” “*only occasionally*,” “*from time to time*,” “*regularly*,” or “*very frequently*”) as a control measure to assess whether familiarity with the target odors influenced typicality judgments. No significant relationship was observed between familiarity and typicality ratings, indicating that individual experience with these odors did not account for the observed patterns (*p* > 0.38, data not shown).

Experiment 2 was restricted to binary mixtures because component detection tasks have limited interpretability for more complex mixtures (e.g., Jinks and Laing 1999). It compared children and adults, as the number of adolescent participants during the second public session was insufficient to constitute a dedicated teenage group. Participants were given a paper questionnaire and four brown vials, each labeled with a three‐digit number and containing either A, B, AB, or AB′. The vials were given to the participants in a random order. An additional vial labeled “Target 1” contained either Odorant A or B (assigned at random). Participants were instructed to first smell “Target 1” and then attempt to detect this odor quality in the other vials. They were informed that the target might be present alongside other odorants or absent altogether, framing the task as an olfactory “Where's Waldo” game (Holy 2014). Participants smelled the target odor before each detection task, thus engaging in a strongly elemental approach. After completing the four detection tasks with “Target 1,” they repeated the same procedure with “Target 2”.

### 2.4 Statistics

All statistical analyses were conducted using R software (R Development Core Team 2011).

In Experiment 1, participants who selected “no odor” when evaluating perceived complexity were excluded from analysis (*n* = 17: 2 children, 8 teenagers, 7 adults). For the complexity evaluation, responses were coded as “one odor” (0) or “more than one odor” (1). These binary data were analyzed using Generalized Estimating Equation modeling (GEE) with subjects as a random factor (geepack package; Hojsgaard et al. 2006). The fixed effects included odor stimulus (AB vs. AB′ or RC vs. RC′), age group, sex, and their interactions with stimulus. The results are presented as the proportion of subjects who responded “one odor.” Response proportions for different stimuli were compared using the Pearson's chi‐squared test with Yates' continuity correction. For typicality data, the responses (“not at all”, “a little”, “moderately”, “a lot”, “tremendously”) were coded from 0 to 4 and analyzed using analysis of variance (ANOVA) with a linear mixed‐effects model and participants as a random factor (nlme package; Pinheiro et al. 2012). The fixed effect was odor stimulus, implemented with interactions of age group, sex, and eating habits. Results are presented as mean typicality scores.

In Experiment 2, the data represent the proportion of children and adults who detected the target in samples. These proportions were analyzed using a GEE model, followed by a comparison of the positive control (“target in target”) with “target in stimuli” using a Wald test statistic. For all analyses, effects were considered significant when *p* < 0.05.

## 3 Results

### 3.1 Experiment 1 ‐ Evaluation of Complexity

#### 3.1.1 In Binary Mixtures

**FIGURE 1:**
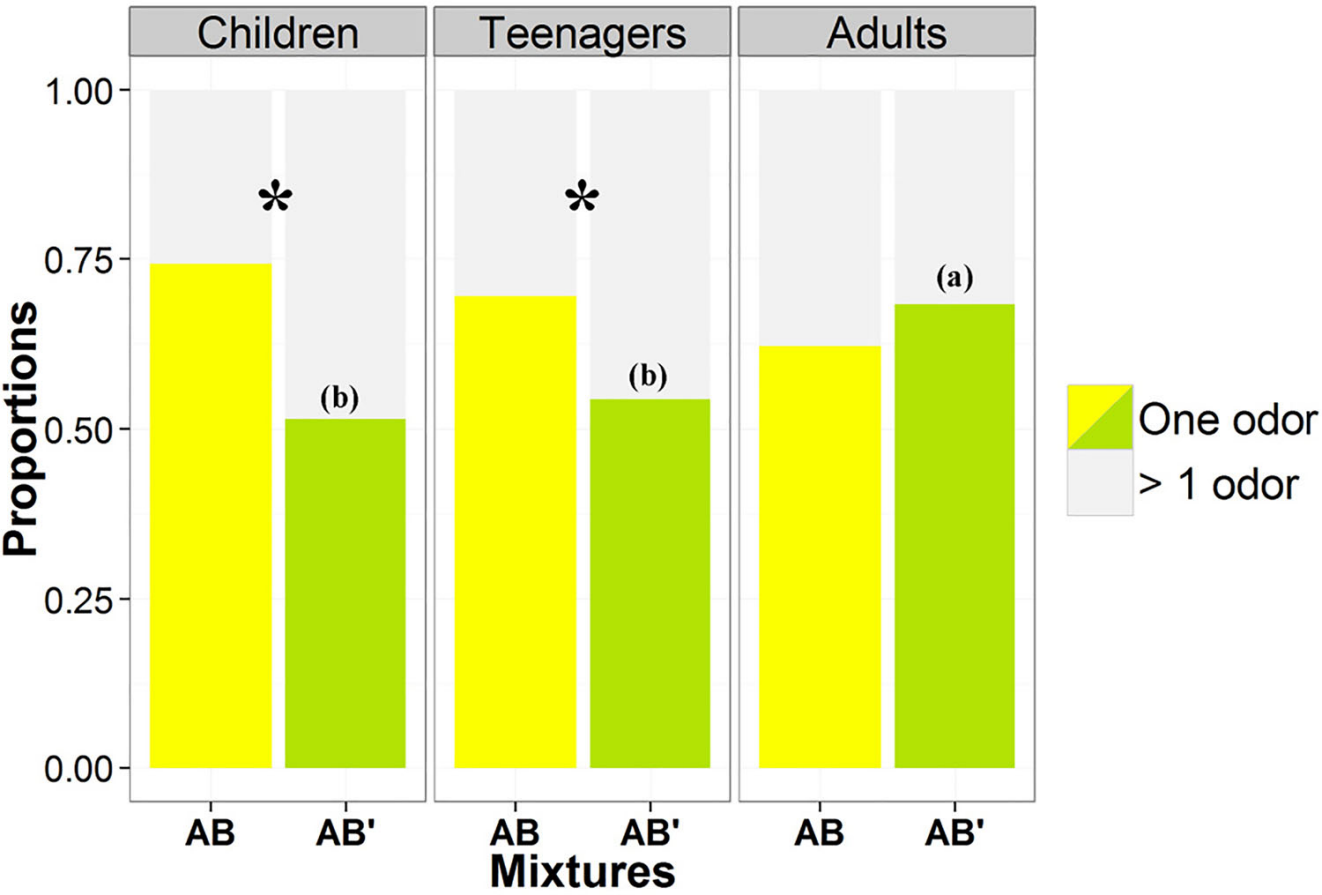
Proportion of children, teenagers, and adults perceiving a single odor when assessing the perceptual complexity of the AB mixture (yellow bars) or the AB′ mixture (green bars). Higher bars indicate lower perceived complexity of the mixture, suggesting a more configural perception. Asterisks indicate significant differences between AB and AB′ within each age group (Pearson *χ* ^2^ test; *p* < 0.05). For AB′, letters in brackets indicate a trend toward an age‐group effect (*p* = 0.076), with adults (a) tending to perceive one odor more frequently than children and teenagers (b).

When evaluating the level of complexity, participants indicated whether they perceived “one odor” or “more than one odor” in the AB or AB′ mixtures. The results revealed a main effect of stimulus (*χ* ^2^(1) = 5.95, *p* = 0.015), with a single odor perceived more frequently in AB (68% of participants) than in AB′ (56%). The model also showed a significant stimulus × age group interaction (*χ* ^2^(2) = 7.67, *p* = 0.024). Among children, 74.3% of participants perceived only one odor in the AB mixture (Figure 1), which was significantly higher than the proportion of 50% who perceived one odor in AB′ (*χ* ^2^(1) = 6.76, *p* = 0.009). Teenagers showed a similar trend, with 68.9% of participants perceiving one odor in AB compared to 52.8% in AB′ (*χ* ^2^(1) = 4.45, *p* = 0.035). In contrast, adults showed no significant difference, with 61.4% of participants reporting one odor in AB and 65.1% in AB′ (*χ* ^2^(1) = 0.43, *p* = 0.5).

The perceived complexity of AB did not differ significantly across age groups (*χ* ^2^(2) = 2.62, *p* = 0.27) (Figure 1). However, the data showed a trend suggesting that adults were more likely than children and teenagers to perceive only one odor in AB′ (65.1% vs. 50% and 52.8%, respectively) (*χ* ^2^(2) = 5.15, *p* = 0.076).

#### 3.1.2 In Senary Mixtures

**FIGURE 2:**
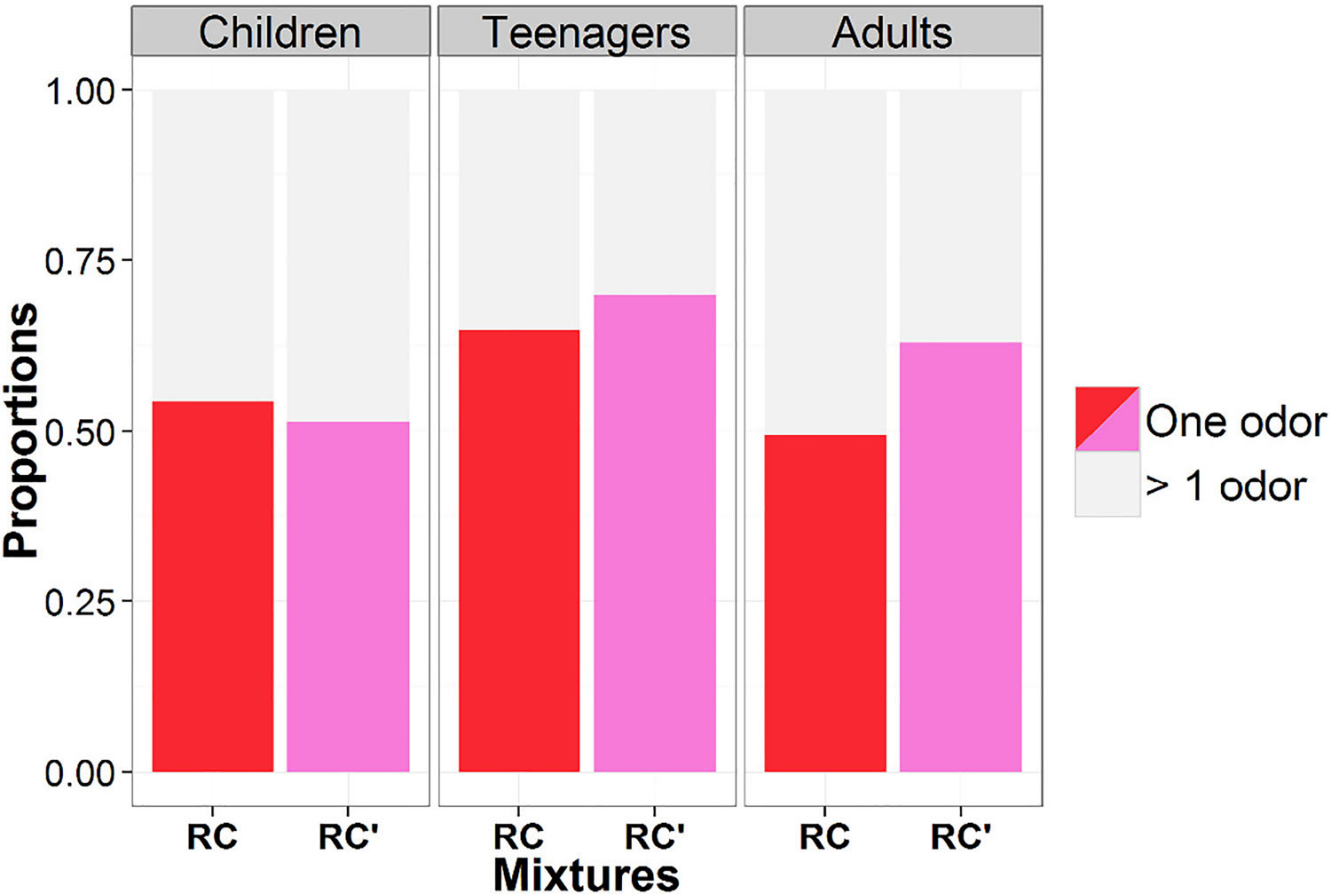
Proportion of children, teenagers and adults perceiving a single odor when assessing the perceptual complexity of the RC (red bars) or RC′ mixtures (pink bars). Higher bars indicate lower perceived complexity of the mixture, suggesting a more configural perception. No significant differences were observed between the two mixtures within any age group (*χ* ^2^ tests, *p* > 0.05).

Participants as a whole did not differentiate between the complexity of the RC and RC′ mixtures: 56% reported perceiving a single odor in RC, compared to 61% in RC′ (*χ* ^2^(1) = 1.68, *p* = 0.2). No significant differences were observed between age groups, as indicated by the non‐significant stimulus × age group interaction (*χ* ^2^(2) = 2.08, *p* = 0.35) (Figure 2).

### 3.2 Experiment 1 ‐ Evaluation of Typicality

#### 3.2.1 In Binary Mixtures

In this task, participants rated how typical the odors of the AB and AB′ mixtures were of “pineapple,” the term that best describes the AB odor quality in adults (Barkat et al. 2012).

**FIGURE 3:**
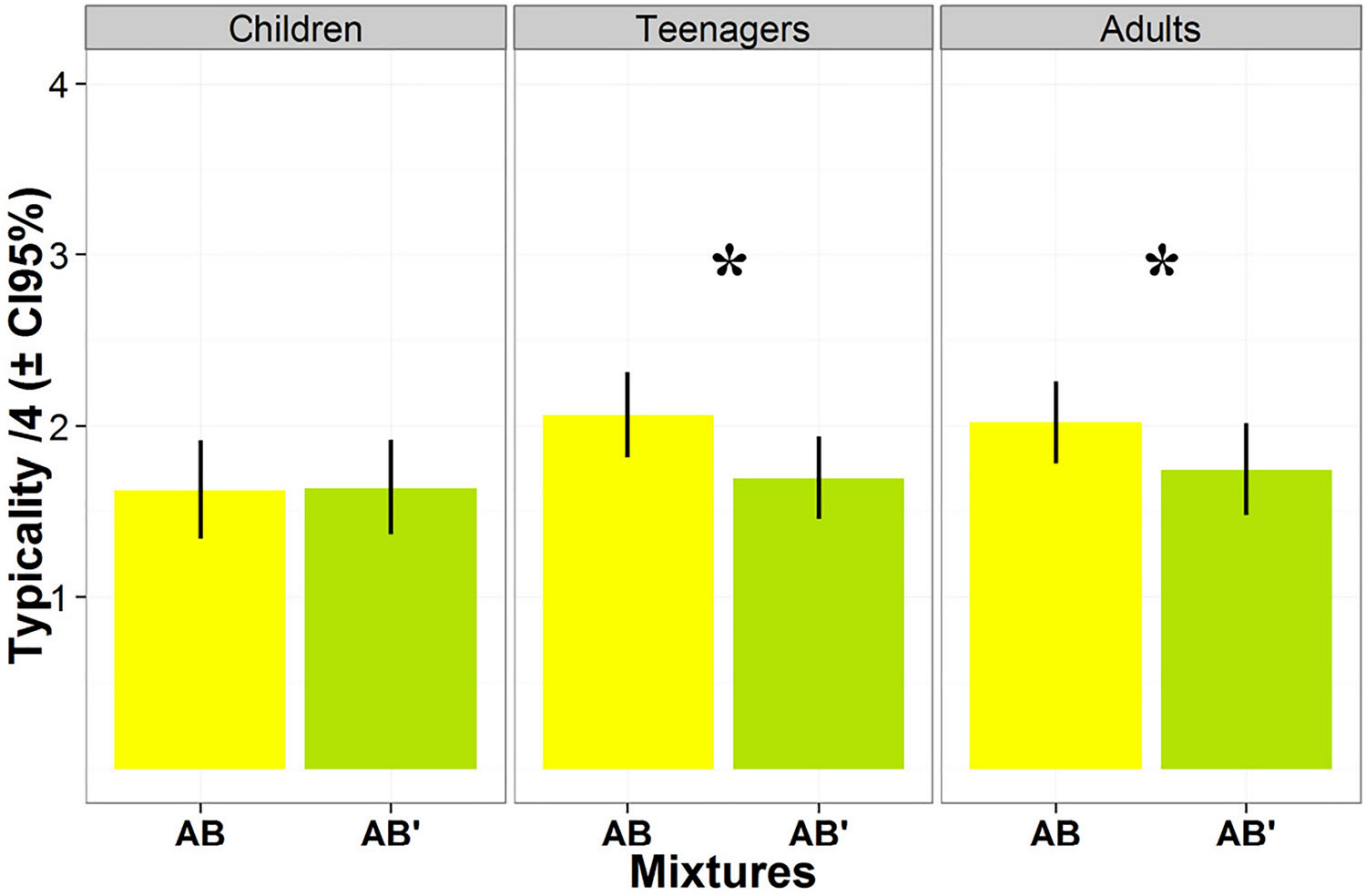
Mean pineapple typicality ratings (± CI 95%) in children, teenagers, and adults for the binary AB mixture (yellow bars) and the AB′ mixture (green bars). Statistical differences between the two mixtures within each age group are indicated by asterisks (paired *t*‐tests; *p* < 0.05).

The data showed a significant main effect of stimulus (*F*(1, 256) = 7.3, *p* = 0.007): AB was rated as more typical of pineapple (mean ± CI 95%: 1.93 ± 0.15) than AB′ (1.70 ± 0.15; paired *t*‐test, *p* = 0.030). Although the stimulus × age group interaction was not significant (*F*(2, 256) = 1.6, *p* = 0.2), descriptive differences across age groups were observed: children did not rate AB as being more pineapple‐like than AB′ (1.63 vs. 1.64, *p* = 0.9) (Figure 3), whereas teenagers and adults rated AB as significantly more typical of pineapple than AB′ (2.1 vs. 1.7, *p* = 0.012, and 2.02 vs. 1.75, *p* = 0.045, respectively).

As a control condition, participants also rated the typicality of AB and AB′ with respect to the odor attribute “Mint.” The low typicality scores (< 0.46 ± 0.09) confirmed the specificity of the “Pineapple” typicality ratings (Table S1). Interestingly, analysis revealed a significant main effect of age group (*F*(2, 256) = 18.1, *p* < 0.0001), with children tending to assign higher mint typicality scores. However, the absence of a significant stimulus × age group interaction (*F*(2, 256) = 0.91, *p* = 0.4) indicated that children generally overestimated typicality scores regardless of stimulus type, and that this overestimation did not account for the observed differences in pineapple and red cordial typicality ratings at this age.

#### 3.2.2 In Senary Mixtures

**FIGURE 4:**
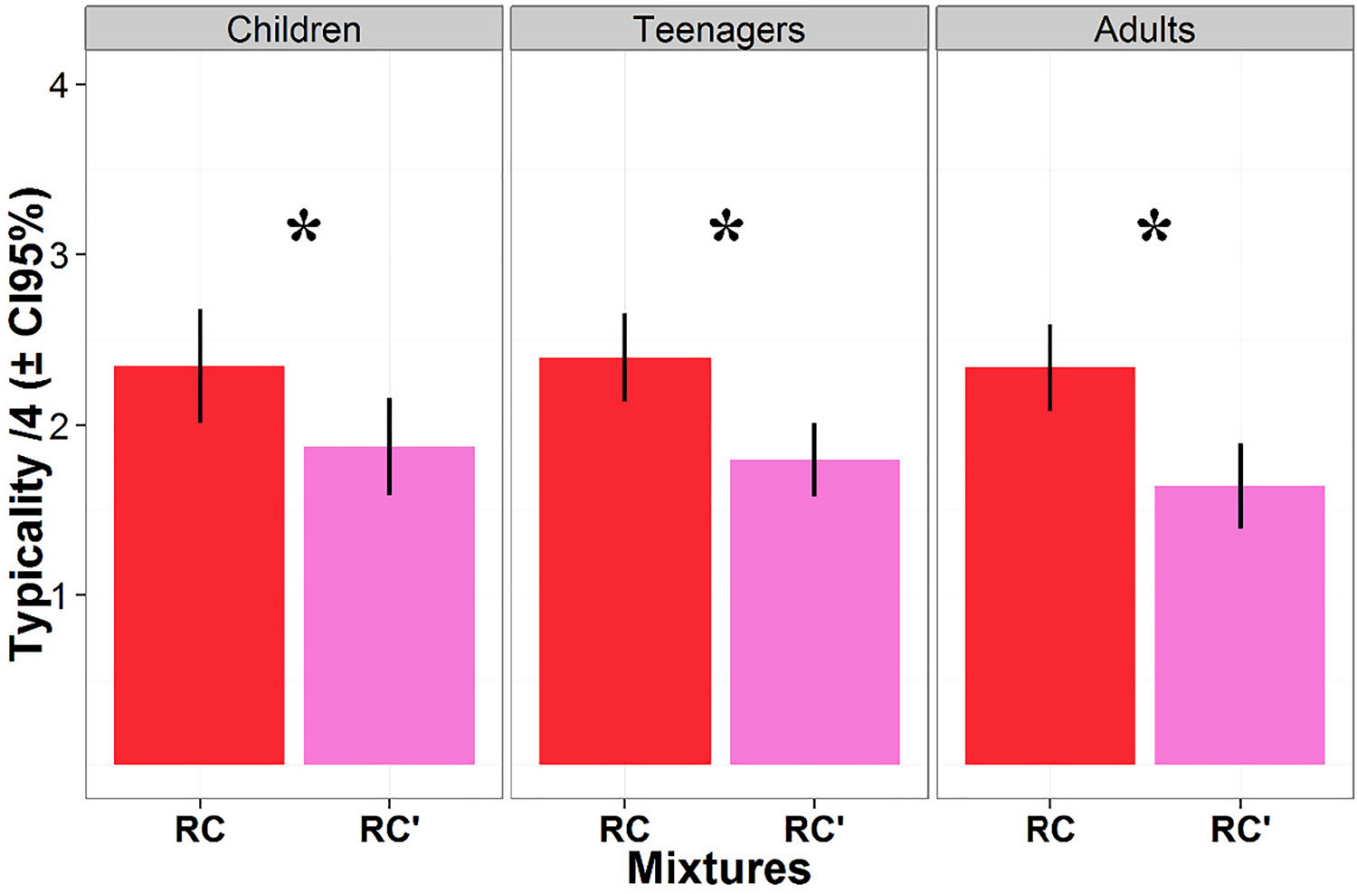
Mean red cordial typicality ratings (± CI 95%) in children, teenagers, and adults for the RC mixture (red bars) and the RC′ mixture (pink bars). Statistical differences between the two mixtures within each age group are indicated by asterisks (paired *t*‐tests; *p* < 0.05).

Participants rated the “red cordial” typicality of the two senary mixtures RC and RC′ (“red cordial” being the term most commonly used to describe the odor quality of the RC mixture in adults; Le Berre, Thomas‐Danguin, et al. 2008). The results showed a significant main effect of stimulus (*F*(1, 256) = 39.7, *p* < 0.0001): RC was rated as more typical of red cordial (2.36 ± 0.28) than RC′ (1.76 ± 0.21). This difference was consistent across age groups (Figure 4), with no significant stimulus × age group interaction (*F*(2, 256) = 0.4, *p* = 0.6).

For the control label “lemon,” mean typicality scores were much lower (< 0.51 ± 0.1; Table S1), with no significant effect of stimulus (*F*(1, 256) = 2.86, *p* = 0.09) or stimulus × age group interaction (*F*(2, 256) = 1.52, *p* = 0.22)).

Overall, the results of Experiment 1 indicate that the perception of the senary mixtures remained consistent across developmental stages. However, differences emerged between children and adults in both the perceived complexity of binary mixtures and their typicality ratings for pineapple.

### 3.3 Experiment 2 – Detection of Target Odorants in Mixtures

In this experiment, children and adults were asked to identify target Odorants A and B in four presented stimuli: A, B, AB, and AB′. The data were analyzed separately for each age group and each target odorant.

**FIGURE 5:**
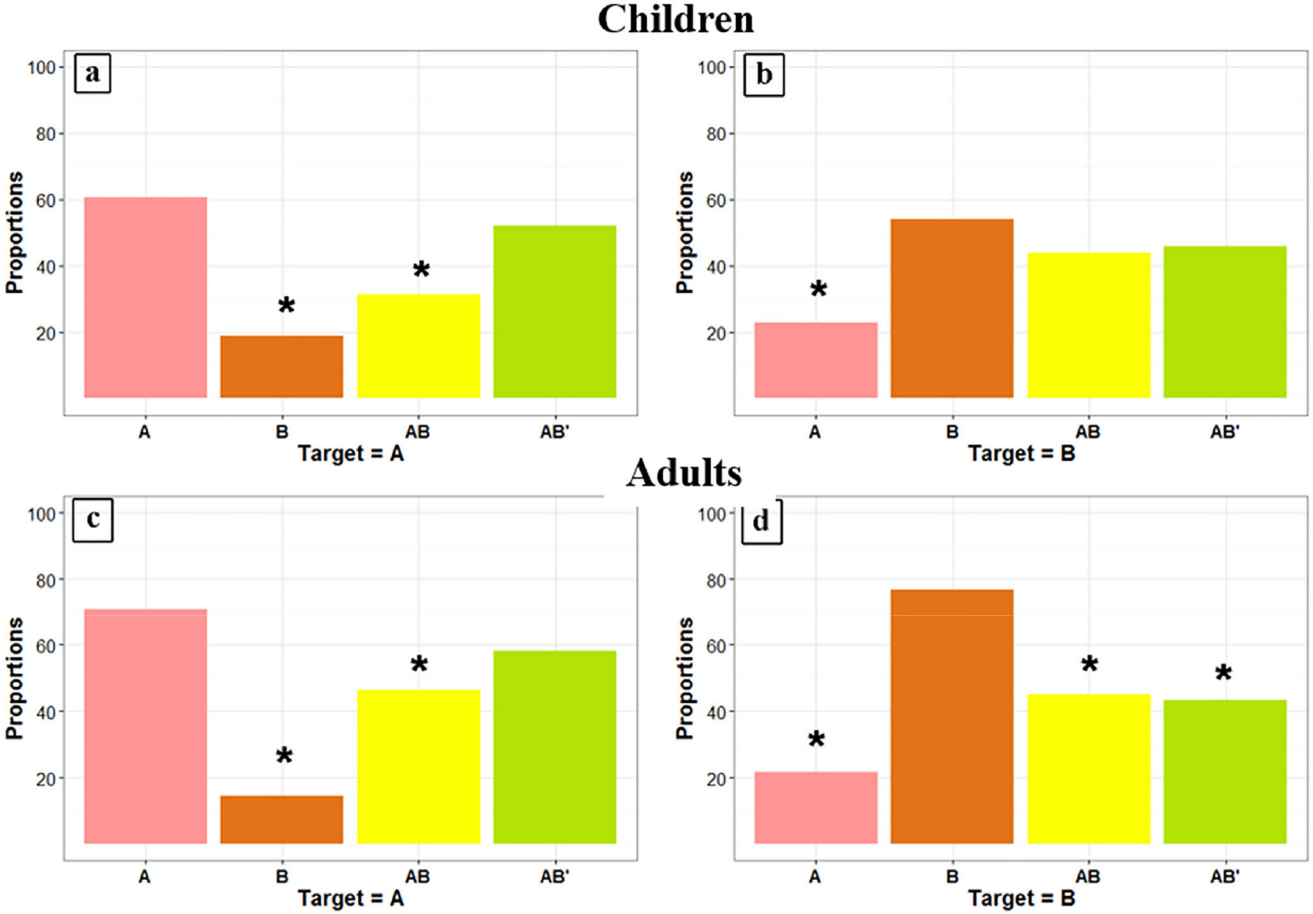
Proportion of children detecting the target Odorant A (panel a) or B (panel b), and of adults detecting the Odorant A (panel c) or B (panel d) in the individual odorants A or B, and in the AB and AB′ mixtures. Statistical differences compared to the positive control (i.e., A in A or B in B) are indicated by asterisks (Wald tests; *p* < 0.05).

For children, detection rates for Odorant A differed significantly across stimuli (*χ* ^2^(3) = 20.2, *p* = 0.0002). While 60.4% of children correctly identified A in the control stimulus A, a significantly lower proportion identified A in the AB mixture (31.2%, *W* = 8, *p* = 0.0048) and in the negative control B (18.7%, *W* = 16, *p* < 0.0001) (Figure 5a). There was no difference in the proportion of children who detected A in A compared to those who detected A in AB′ (52.1%, *W* = 0.7, *p* = 0.4). For Odorant B, the nature of the stimulus also significantly affected detection rates (*χ* ^2^(3) = 9.91, *p* = 0.019). The target odor B was identified in 54.1% of trials with the control stimulus B (Figure 5b), a proportion equivalent to the detection of B in AB′ (45.8%, *W* < 1.04, *p* > 0.3) and AB (43.75%). Significantly fewer children identified B in stimulus A (21.9%) than in B (*W* = 9.4, *p* = 0.002).

As in children, detection rates in adults differed significantly by stimulus (*χ* ^2^(3) = 40.4, *p* < 0.0001). While 71% of adult participants identified the Odorant A in the control stimulus A, significantly fewer participants detected A in AB (46.4%, *W* = 8.4, *p* = 0.004) or in the negative control B (14.5%, *W* = 38.1, *p* < 0.0001) (Figure 5c). However, detection of A in A did not differ significantly from that in AB′ (58%, *W* = 2.5, *p* = 0.11).

For Odorant B, detection rates also varied by stimulus (*χ* ^2^(3) = 37.6, *p* < 0.0001). A higher proportion of adults correctly detected B in the control stimulus B (76.8%) than in AB (44.9%), AB' (43.5%), or A (21.7%; *W* > 14, *p* < 0.0002) (Figure 5d).

Overall, the results of Experiment 2 highlight age‐group differences in the detection patterns of individual odorants across stimuli, with adults tending to show reduced access to components in mixtures, consistent with a greater tendency toward configural processing in adults than in children.

## 4 Discussion

To our knowledge, this study is the first to investigate whether strategies for processing odor mixtures in humans evolve across development. To this end, we examined the elemental and configural perception of two binary and two senary mixtures, from childhood through adolescence to adulthood. Perception was assessed using three complementary approaches: typicality rating (which favors configural processing), component detection (which promotes elemental processing), and perceptual complexity evaluation (which is relatively neutral with respect to processing strategy). While none of these tasks provides a direct readout of processing mode in isolation, together they offer convergent indices of elemental and configural perception.

Regarding the senary mixtures RC and RC′, results from Experiment 1 showed that participants perceived both mixtures as having similar levels of perceptual complexity, but rated RC as more typical of the red cordial odor than RC′. These results confirm the configural status of RC, which is consistent with previous findings in human adults and newborn rabbits (Le Berre, Thomas‐Danguin, et al. 2008; Le Berre et al. 2010; Barkat et al. 2012; Sinding et al. 2013; Romagny et al. 2014). In Experiment 1, neither perceived complexity nor typicality ratings varied across age groups, suggesting developmental stability in the perception of this six‐component mixture. The RC mixture has previously been shown to maintain its configural quality even after exposure of participants to its individual components (Le Berre, Thomas‐Danguin, et al. 2008). Such perceptual stability may result from the chemical complexity of this mixture, which likely exceeds the limits of human sensory and cognitive elemental processing, that is, typically three to four components (Laing and Francis 1989; Livermore and Laing 1998). In such cases, configural perception may emerge spontaneously and remain unaffected by age or learning (Jinks and Laing 1999; Le Berre, Thomas‐Danguin, et al. 2008). Although RC′ was rated as less typical of the red cordial odor, it was still mostly perceived as a single odor across all age groups (Experiment 1). This may reflect an overshadowing effect within the mixture, whereby one component becomes perceptually dominant. Such an effect is consistent with findings from chemically modified versions of the RC mixture, in which altering component proportions led to a perceptual shift toward one of the mixture's components (Romagny et al. 2018).

By contrast, developmental differences were observed with the simpler binary mixtures AB and AB′, which are respectively known to be processed configurally and elementally (Le Berre, Thomas‐Danguin, et al. 2008; Le Berre et al. 2010; Sinding et al. 2011). Across ages, results from Experiment 1 showed that AB was mostly perceived as less complex than AB′, consistent with previous reports supporting its configural perception in adults, where it evokes the configural pineapple odor quality (Le Berre, Thomas‐Danguin, et al. 2008; Le Berre et al. 2010). By comparison, the AB′ mixture, which has a different component ratio, was perceived more elementally in our study, as previously shown even for minor variations in component concentrations (Le Berre, Thomas‐Danguin, et al. 2008; Sinding 2012). However, our results also highlighted developmental differences in the processing of these binary odor mixtures. In Experiment 1, most children rated the AB mixture as containing a single odor, but this percept did not correspond to the expected pineapple odor quality of the configuration. Indeed, in Experiment 2, children more frequently identified the caramel odor associated with Component B when evaluating the AB mixture, suggesting greater access to this component. This interpretation is consistent with the typicality results from Experiment 1, which suggested that AB evoked the pineapple quality less strongly in children than in teenagers and adults, although the stimulus × age‐group interaction was not significant. For the AB′ mixture, children rated the perceived complexity as higher in Experiment 1 and were able to detect both Components A and B in the mixture in Experiment 2. In contrast, adults rated AB and AB′ as similarly complex, but perceived AB as more typical of pineapple (Experiment 1). Taken together, these convergent results suggest a more elemental mode of processing in children, whereas adults showed a stronger tendency to integrate mixture components into a configural percept.

Detection data from Experiment 2 only partially supported the hypothesis that access to individual components would be lower in the more configurally perceived AB than in the more elementally perceived AB′ mixture. This pattern was clearer for Odorant A. For Odorant B, the main between‐group difference was observed for the control stimulus B, with adults identifying this odorant more frequently than children. Nevertheless, the decrease in B detection from the pure B stimulus to the AB and AB′ mixtures was greater in adults than in children, suggesting reduced access to this component in mixture contexts, consistent with a greater tendency toward configural processing in adults. This interpretation should also be considered in light of the task demands of Experiment 2. Because component detection explicitly encourages participants to search for individual odor qualities, it may have attenuated the expression of configural percepts and thereby reduced the observable contrast between AB and AB′. Teenagers appeared to exhibit an intermediate profile in Experiment 1. Like adults, they rated AB as more typical of pineapple than AB′, but like children, they still perceived AB′ as more complex than AB. This pattern is consistent with the possibility that configural processing continues to strengthen throughout childhood and adolescence.

Similar developmental trajectories have been described in non‐human mammals using the same odor mixtures. In newborn rabbits, the AB mixture initially evokes a weak configural percept (at postnatal Day 2), but becomes robust configural by postnatal Day 9. In contrast, the AB′ mixture, initially perceived as purely elemental, requires a more advanced developmental stage to engage a weak configural processing (postnatal Day 24) (Coureaud et al. 2020). These cross‐species comparisons should nevertheless be interpreted with caution. The present study relied on explicit judgments, whereas animal studies use nonverbal behavioral indices. Although both approaches broadly converge in distinguishing elemental from configural processing (Sinding et al. 2013), explicit measures are more susceptible to the influence of language, attention, and response strategies, particularly when comparing different age groups.

In the AB′ mixture, children were more likely than adults to extract components’ odor features. Similar extraction abilities have been reported in visual perception, where younger individuals (aged 6–8) more readily identify facial features (components), whereas older children (10 years) and adults rely more on the spatial relationships between features (configuration) (Mondloch et al. 2002). This suggests that, in both vision and olfaction, younger individuals possess enhanced element extraction abilities for less complex stimuli compared to adults, supporting the hypothesis that elemental perception serves as the primary processing strategy in early human development (Younger and Cohen 1986; Scott 2006; Wakui et al. 2013). Thus, development may reduce the perceived complexity of certain stimuli by minimizing the number of detected components while favoring more integrative, configural strategies. In olfaction, this developmental shift could help preserve the biological relevance of certain odor mixtures by maintaining their salient configural qualities, while also simplifying the olfactory environment for developing individuals by reducing cognitive load and limiting the perception of individual components (Gheusi and Lledo 2014).

This developmental pattern was particularly apparent for the AB mixture, where configural processing appeared more prevalent in teenagers than in younger children. Teenagers rated AB as more typical of pineapple than children, suggesting a greater expression of the configural percept in this group. This is also consistent with findings in vision, where holistic face representations begin to emerge around the age of 10. Indeed, for face perception, older children (aged 9–11) showed an intermediate pattern as well. They tended toward holistic processing of second‐order faces, meaning that, in addition to basic facial features, they integrated subtle spatial relationships between these features into a unified percept, similar to adults. Nevertheless, they exhibited parallel visual processing in other experimental conditions, as observed in younger children (aged 6–8) (Joseph et al. 2015). This intermediate profile in teenagers may also reflect the substantial individual differences in the development of configural perception observed throughout childhood and adolescence, as reported in the domain of face perception (Petrakova et al. 2018).

In conclusion, this study supports the idea that the processing of complex olfactory stimuli in humans evolves during development. While chemically complex odor mixtures appear to be processed configurally regardless of age, simpler mixtures seem to be more sensitive to developmental differences in perceptual strategy. This contrast suggests that configural processing may emerge at different developmental stages depending not only on stimulus properties, but also on the maturation of cognitive processes. Highly complex mixtures may promote earlier unified percepts because their components are more difficult to access individually, whereas simpler mixtures may remain open to elemental processing for longer and may require greater perceptual experience or top‐down integration to be processed configurally. Overall, these findings are consistent with a progressive shift from a more elemental toward a more configural mode of processing with increasing age. Olfaction therefore offers a promising model for studying how perceptual organization develops over time. However, these conclusions should be interpreted in light of the specific mixtures tested here. AB/AB′ and RC/RC′ were selected as well‐characterized models of elemental and configural processing, but developmental effects may also depend on familiarity, ecological relevance, and the perceptual salience of particular odor qualities. The public outreach setting in which the data were collected should also be considered; despite efforts to standardize procedures, it may have introduced additional variability in attention, task engagement, or environmental context, particularly among younger participants. Future research should explore this developmental evolution further through longitudinal designs, by testing additional mixtures of varying complexity and including broader age ranges to better characterize developmental transitions from elemental to configural perception.

## Conflicts of Interest

The authors declare no conflicts of interest.

## Supporting information




**Supplementary Table S1**: Mean (± SD) typicality ratings for the control odor labels across age groups and tested stimuli. For the binary mixtures (AB and AB′), the control label was *mint*, whereas for the senary mixtures (RC and RC′), the control label was *lemon*. Low ratings across conditions confirmed the specificity of the target typicality judgments.

## Data Availability

The data supporting the findings of this study are available from the corresponding author upon reasonable request.

## References

[dev70188-bib-0001] Barkat, S. , E. Le Berre , G. Coureaud , G. Sicard , and T. Thomas‐Danguin . 2012. “Perceptual Blending in Odor Mixtures Depends on the Nature of Odorants and Human Olfactory Expertise.” Chemical Senses 37: 159–166. 10.1093/chemse/bjr086. 21873604

[dev70188-bib-0002] Chandra, S. , and B. H. Smith . 1998. “An Analysis of Synthetic Processing of Odor Mixtures in the Honeybee (*Apis mellifera*).” Journal of Experimental Biology 201: 3113–3121. 9787131 10.1242/jeb.201.22.3113

[dev70188-bib-0003] Cohen Kadosh, K. , M. H. Johnson , R. N. A. Henson , F. Dick , and S.‐J. Blakemore . 2013. “Differential Face‐Network Adaptation in Children, Adolescents and Adults.” Neuroimage 69: 11–20. 10.1016/j.neuroimage.2012.11.060. 23231884

[dev70188-bib-0004] Coureaud, G. , Y. Hamdani , B. Schaal , and T. Thomas‐Danguin . 2009. “Elemental and Configural Processing of Odour Mixtures in the Newborn Rabbit.” Journal of Experimental Biology 212: 2525–2531. 10.1242/jeb.032235. 19648396

[dev70188-bib-0005] Coureaud, G. , C. Letagneau , T. Thomas‐Danguin , and S. Romagny . 2020. “Developmental Changes in Elemental and Configural Perception of Odor Mixtures in Young Rabbits.” Developmental Psychobiology 62: 471–483. 10.1002/dev.21929. 31628769

[dev70188-bib-0006] Coureaud, G. , B. Schaal , R. Hudson , P. Orgeur , and P. Coudert . 2002. “Transnatal Olfactory Continuity in the Rabbit: Behavioral Evidence and Short‐Term Consequence of Its Disruption.” Developmental Psychobiology 40: 372–390. 10.1002/dev.10038. 12115295

[dev70188-bib-0007] Coureaud, G. , T. Thomas‐Danguin , F. Datiche , D. A. Wilson , and G. Ferreira . 2014. “Differential Memory Persistence of Odor Mixture and Components in Newborn Rabbits: Competition Between the Whole and Its Parts.” Frontiers in Behavioral Neuroscience 8: 1–10. 10.3389/fnbeh.2014.00211. 24982622 PMC4059275

[dev70188-bib-0008] Coureaud, G. , T. Thomas‐Danguin , E. Le Berre , and B. Schaal . 2008. “Perception of Odor Blending Mixtures in the Newborn Rabbit.” Physiology & Behavior 95: 194–199. 10.1016/j.physbeh.2008.05.018. 18586286

[dev70188-bib-0009] Coureaud, G. , T. Thomas‐Danguin , D. A. Wilson , and G. Ferreira . 2014. “Neonatal Representation of Odour Objects: Distinct Memories of the Whole and Its Parts.” Proceedings of the Royal Society B: Biological Sciences 281: 20133319. 10.1098/rspb.2013.3319. PMC4100496 24990670

[dev70188-bib-0045] Coureaud, G. , T. Thomas‐Danguin , J.‐C. Sandoz , and D. A. Wilson . 2022. “Biological constraints on configural odour mixtureperception.” Journal of Experimental Biology 225: jeb242274. 10.1242/jeb.242274. 35285471 PMC8996812

[dev70188-bib-0010] Deisig, N. , H. Lachnit , M. Giurfa , and F. Hellstern . 2001. “Configural Olfactory Learning in Honeybees : Negative and Positive Patterning Discrimination.” Learning & Memory 8, no. 2: 70–78. 10.1101/lm.38301. 11274252 PMC311365

[dev70188-bib-0011] Derby, C. D. , M. Hutson , A. Livermore , and W. H. Lynn . 1996. “Generalization Among Related Complex Odorant Mixtures and Their Components: Analysis of Olfactory Perception in the Spiny Lobster.” Physiology & Behavior 60: 87–95. 8804647 10.1016/0031-9384(95)02237-6

[dev70188-bib-0012] R Development Core Team . 2011. “R: A Language and Environment for Statistical Computing.” https://www.r‐project.org/.

[dev70188-bib-0013] Gheusi, G. , and P.‐M. Lledo . 2014. “Adult Neurogenesis in the Olfactory System Shapes Odor Memory and Perception.” In Progress in Brain Research: Odor Memory and Perception, edited by E. Barkai and D. A. Wilson , 157–175. Elsevier B.V.. 10.1016/B978-0-444-63350-7.00006-1. 24767482

[dev70188-bib-0014] Gottlieb, G. 1971. “Ontogenesis of Sensory Function in Birds and Mammals.” In The Biopsychology of Development, edited by E. Tobach L. R. Aronson , and E. Shaw , 67–128, Academic Press.

[dev70188-bib-0015] Gottlieb, G. 1983. “The Psychobiological Approach to Developmental issues.” In *Handbook of Child Psychobiology (*Vol. *2)* . 4th ed., edited by M. M. Haith and J. J. Campos , 1–26, Wiley & Sons.

[dev70188-bib-0016] Hojsgaard, S. , U. Halekoh , and J. Yan . 2006. “The R Package Geepack for Generalized Estimating Equations.” Journal of Statistical Software 15: 1–11.

[dev70188-bib-0017] Holy, T. E. 2014. “Scenting Waldo: Analyzing Olfactory Scenes.” Nature Neuroscience 17: 1144–1145. 10.1038/nn.3796. 25157510

[dev70188-bib-0018] Jinks, A. , and D. G. Laing . 1999. “A Limit in the Processing of Components in Odour Mixtures.” Perception 28: 395–404. 10.1068/p2898. 10615476

[dev70188-bib-0019] Joseph, J. E. , M. D. DiBartolo , and R. S. Bhatt . 2015. “Developmental Changes in Analytic and Holistic Processes in Face Perception.” Frontiers in Psychology 6: 1165. 10.3389/fpsyg.2015.01165. 26300838 PMC4528094

[dev70188-bib-0020] Kay, L. M. , T. Crk , and J. Thorngate . 2005. “A Redefinition of Odor Mixture Quality.” Behavioral Neuroscience 119: 726–733. 10.1037/0735-7044.119.3.726. 15998193

[dev70188-bib-0021] Laing, D. G. , and G. W. Francis . 1989. “The Capacity of Humans to Identify Odors in Mixtures.” Physiology & Behavior 46: 809–814. 10.1016/0031-9384(89)90041-3. 2628992

[dev70188-bib-0022] Le Berre, E. , N. Béno , A. Ishii , C. Chabanet , P. Etiévant , and T. Thomas‐Danguin . 2008. “Just Noticeable Differences in Component Concentrations Modify the Odor Quality of a Blending Mixture.” Chemical Senses 33: 389–395. 10.1093/chemse/bjn006. 18304991

[dev70188-bib-0023] Le Berre, E. , E. Jarmuzek , N. Béno , P. Etiévant , J. Prescott , and T. Thomas‐Danguin . 2010. “Learning Influences the Perception of Odor Mixtures.” Chemosensory Perception 3: 156–166. 10.1007/s12078-010-9076-y.

[dev70188-bib-0024] Le Berre, E. , T. Thomas‐Danguin , N. Béno , G. Coureaud , P. Etiévant , and J. Prescott . 2008. “Perceptual Processing Strategy and Exposure Influence the Perception of Odor Mixtures.” Chemical Senses 33: 193–199. 10.1093/chemse/bjm080. 18071197

[dev70188-bib-0025] Livermore, A. , and D. G. Laing . 1996. “Influence of Training and Experience on the Perception of Multicomponent Odor Mixtures.” Journal of Experimental Psychology: Human Perception and Performance 22: 267–277. 10.1037/0096-1523.22.2.267. 8934843

[dev70188-bib-0026] Livermore, A. , and D. G. Laing . 1998. “The Influence of Chemical Complexity on the Perception of Multicomponent Odor Mixtures.” Perception and Psychophysics 60: 650–661. 10.3758/BF03206052. 9628996

[dev70188-bib-0027] Mondloch, C. J. , R. L. Grand , and D. Maurer . 2002. “Configural Face Processing Develops More Slowly Than Featural Face Processing.” Perception 31: 553–566. 10.1068/p3339. 12044096

[dev70188-bib-0028] Petrakova, A. , W. Sommer , M. Junge , and A. Hildebrandt . 2018. “Configural Face Perception in Childhood and Adolescence: An Individual Differences Approach.” Acta Psychologica 188: 148–176. 10.1016/j.actpsy.2018.06.005. 29940535

[dev70188-bib-0029] Pinheiro, J. , D. Bates , S. DebRoy , and D. Sarkar , and R Development Core Team . 2012. “nlme: Linear and Nonlinear Mixed Effects Models.” https://cran.r‐project.org/web/packages/nlme/index.html.

[dev70188-bib-0030] Rokni, D. , and V. N. Murthy . 2014. “Analysis and Synthesis in Olfaction.” ACS Chemical Neuroscience 5: 870–872. 10.1021/cn500199n. 25238650 PMC4334211

[dev70188-bib-0031] Romagny, S. , G. Coureaud , and T. Thomas‐Danguin . 2018. “Key Odorants or Key Associations? Insights Into Elemental and Configural Odour Processing.” Flavour Fragr J 33: 97–105. 10.1002/ffj.3429.

[dev70188-bib-0032] Romagny, S. , A.‐S. Darmaillacq , M. Guibé , C. Bellanger , and L. Dickel . 2012. “Feel, Smell and See in an Egg: Emergence of Perception and Learning in an Immature Invertebrate, the Cuttlefish Embryo.” Journal of Experimental Biology 215: 4125–4130. 10.1242/jeb.078295. 23136152

[dev70188-bib-0033] Romagny, S. , T. Thomas‐Danguin , and G. Coureaud . 2014. “Newborn Rabbit Perception of 6‐Odorant Mixtures Depends on Configural Processing and Number of Familiar Elements.” PLoS ONE 9: e107560. 10.1371/journal.pone.0107560. 25248149 PMC4172776

[dev70188-bib-0034] Schaal, B. 2000. “Human Foetuses Learn Odours From Their Pregnant Mother's Diet.” Chemical Senses 25: 729–737. 10.1093/chemse/25.6.729. 11114151

[dev70188-bib-0035] Scott, L. S. 2006. “Featural and Configural Face Processing in Adults and Infants: A Behavioral and Electrophysiological Investigation.” Perception 35: 1107–1128. 10.1068/p5493. 17076069

[dev70188-bib-0036] Sinding, C. 2012. “Perception Des Mélanges D'odeurs : Etude Comportementale et Psychophysique Chez Le Mammifère Nouveau‐né et L'Homme.” PhD thesis, Université de Bourgogne.

[dev70188-bib-0037] Sinding, C. , G. Coureaud , B. Bervialle , C. Martin , B. Schaal , and T. Thomas‐Danguin . 2015. “Experience Shapes Our Odor Perception but Depends on the Initial Perceptual Processing of the Stimulus.” Attention, Perception, & Psychophysics 77: 1794‑1806. 10.3758/s13414-015-0883-8. 25832188

[dev70188-bib-0038] Sinding, C. , T. Thomas‐Danguin , A. Chambault , et al. 2013. “Rabbit Neonates and Human Adults Perceive a Blending 6‐Component Odor Mixture in a Comparable Manner.” PLoS ONE 8: 1–12. 10.1371/journal.pone.0053534. PMC3547025 23341948

[dev70188-bib-0039] Sinding, C. , T. Thomas‐Danguin , G. Crepeaux , B. Schaal , and G. Coureaud . 2011. “Experience Influences Elemental and Configural Perception of Certain Binary Odour Mixtures in Newborn Rabbits.” Journal of Experimental Biology 214: 4171–4178. 10.1242/jeb.063610. 22116759

[dev70188-bib-0040] Thomas‐Danguin, T. , E. Le Berre , S. Barkat , G. Coureaud , and G. Sicard . 2007. “Evidence for Odor Blending in Odorant Mixtures.” Paper presented at 29th Annual Meeting of the Association for Chemoreception Sciences, Sarasota, FL, April 25–29.

[dev70188-bib-0041] Thomas‐Danguin, T. , C. Sinding , S. Romagny , et al. 2014. “The Perception of Odor Objects in Everyday Life: A Review on the Processing of Odor Mixtures.” Frontiers in Psychology 5: 1–18. 10.3389/fpsyg.2014.00504. 24917831 PMC4040494

[dev70188-bib-0042] Wakui, E. , M. Jüttner , D. Petters , S. Kaur , J. E. Hummel , and J. Davidoff . 2013. “Earlier Development of Analytical Than Holistic Object Recognition in Adolescence.” PLoS ONE 8: e61041. 10.1371/journal.pone.0061041. 23577188 PMC3618112

[dev70188-bib-0043] Wilson, D. A. , and R. J. Stevenson . 2003. “Olfactory Perceptual Learning: The Critical Role of Memory in Odor Discrimination.” Neuroscience and Biobehavioral Reviews 27: 307–328. 10.1016/S0149-7634(03)00050-2. 12946684

[dev70188-bib-0044] Younger, B. A. , and L. B. Cohen . 1986. “Developmental Change in Infants' Perception of Correlations Among Attributes.” Child Development 57: 803–815. 3720405

