# Supplementary material for: Developmental Switch From Elemental to Configural Processing of Odor Mixtures in Humans
Source: Dev Psychobiol. 2026 Aug 5;68(5):e70188. doi: 10.1002/dev.70188 (PMC13439156; doi:10.1002/dev.70188)
Supplement: Supplementary file 1 [file DEV-68-e70188-s001.docx]

**Supplementary Materials**

**Supplementary Table S1:** Mean (± SD) typicality ratings for the control odor labels across age groups and tested stimuli. For the binary mixtures (AB and AB′), the control label was *mint*, whereas for the senary mixtures (RC and RC′), the control label was *lemon*. Low ratings across conditions confirmed the specificity of the target typicality judgments.

| **Age group** | **Tested stimulus** | **Mean control typicality rating** | **SD** |
| --- | --- | --- | --- |
| Adults | AB | 0.12 | 0.33 |
| Children | AB | 0.50 | 0.76 |
| Teenagers | AB | 0.43 | 0.70 |
| Adults | AB′ | 0.14 | 0.39 |
| Children | AB′ | 0.71 | 0.93 |
| Teenagers | AB′ | 0.56 | 0.78 |
| Adults | RC | 0.30 | 0.56 |
| Children | RC | 0.71 | 0.90 |
| Teenagers | RC | 0.30 | 0.66 |
| Adults | RC′ | 0.25 | 0.51 |
| Children | RC′ | 0.89 | 1.11 |
| Teenagers | RC′ | 0.46 | 0.86 |
